# The Relationship between Trait Emotional Intelligence and Interaction with Ostracized Others' Retaliation

**DOI:** 10.1371/journal.pone.0077579

**Published:** 2013-10-23

**Authors:** Yuki Nozaki, Masuo Koyasu

**Affiliations:** 1 Graduate School of Education, Kyoto University, Kyoto, Japan; 2 Japan Society for the Promotion of Science, Tokyo, Japan; George Mason University / Krasnow Institute for Advanced Study, United States of America

## Abstract

**Background:**

Regulation of emotions in others is distinct from other activities related to trait emotional intelligence in that only such behavior can directly change other people's psychological states. Although emotional intelligence has generally been associated with prosociality, emotionally intelligent people may manipulate others' behaviors to suit their own interests using high-level capabilities to read and manage the emotions of others. This study investigated how trait emotional intelligence was related to interacting with ostracized others who attempt retaliation.

**Method:**

We experimentally manipulated whether two people were simultaneously ostracized or not by using an online ball-tossing game called Cyberball. Eighty university students participated in Cyberball for manipulating ostracism and a “recommendation game,” a variation of the ultimatum game for assessing how to interact with others who attempt retaliation, with four participants. After the recommendation game, participants rated their intention to retaliate during the game.

**Results:**

People with higher interpersonal emotional intelligence were more likely to recommend that the ostracized other should inhibit retaliation and maximize additional rewards when they have a weaker intention to retaliate. However, they were more likely to recommend that the ostracized other should retaliate against the ostracizers when they have a stronger intention to retaliate.

**Conclusion:**

This is the first laboratory study that empirically reveals that people with high interpersonal emotional intelligence influence others' emotions based on their own goals contrary to the general view. Trait emotional intelligence itself is neither positive nor negative, but it can facilitate interpersonal behaviors for achieving goals. Our study offers valuable contributions for the refinement of the trait emotional intelligence concept in the respect of its social function.

## Introduction

Emotional intelligence (EI) refers to individual differences in the extent to which one appropriately appraises and regulates self-related and other-related emotions [1], [2]. EI has generally been associated with prosociality [3]. High levels of it are associated with better interpersonal relationships [4]–[6]. However, recent studies have suggested that EI can facilitate not only socially valued behavior [7]–[9]. For example, emotionally intelligent people may manipulate others' behaviors to suit their own interests rather than achieving general prosocial outcomes using high-level capabilities to read and manage the emotions of others [7]. This paper investigates the possibility that emotionally intelligent people influence the emotions of others based on their own goals.

EI is classified in one of two ways: trait and ability EI [10]. Trait EI is conceived as a constellation of emotional self-perceptions located at the lower levels of personality hierarchies [11]. This concept is measured through self-report questionnaires to tap typical performances. Previous studies have demonstrated incremental validity to predict a number of emotional reactions and achievements over and above such established constructs as Big Five [12]–[14]. On the other hand, ability EI, which is conceived as a distinct group of mental abilities in emotional functioning [1], [15], is measured through maximum-performance tests (IQ-like tests).

Both EI theories regard EI as including several domains of specific skills. The trait EI theory has usually considered the regulation of emotions in others as a distinct domain and distinguishes between intrapersonal (e.g., appraisal and regulation of emotions in the self) and interpersonal domains (e.g., appraisal and regulation of emotions in others) [10], [16]. In contrast, the ability EI theory has not sharply separated the intrapersonal and interpersonal domains as subcomponents of EI [17], [18]. Since previous studies argue that the regulation of emotions in the self and others should be clearly distinguished [19], we focus on trait EI in this study.

### Trait EI and regulation of emotions in others

People try to regulate other people's emotions or influence how others express them in interpersonal situations [20]. When people confront interpersonal problems, they must appraise and regulate others' emotions as well as their own emotions for constructive outcomes. The regulation of emotions in others is distinct from other activities related to trait EI (e.g., regulation of emotions in the self and appraisal of others' emotions) in that only such behavior can directly change other people's psychological states. Thus, it is important to reveal how individual differences in trait EI are reflected in behavior for regulating others' emotions when they experience interpersonal problems.

Previous laboratory studies have revealed how people with high trait EI recognize others' emotions [21], [22], regulate their own negative emotions in personal stressful situations [23], [24], and inhibit retaliation after experiencing ostracism [25]. However, to the best of our knowledge, no previous study has investigated how emotionally intelligent people behave for regulating others' emotions on the basis of rapid and accurate emotion recognition. Thus, we must assess the regulation of emotions in others with a behavioral measure and investigate the relationship between trait EI and this behavior in given interpersonal situations to clarify trait EI's social functions.

There are two possibilities about how emotionally intelligent people behave for regulating others' emotions. One is that they regulate others' emotions so that others act rationally. Previous studies suggested that emotionally intelligent people made better decisions in a financial gambling task even though an irrelevant affective cue appeared [26] and preferred a deliberate decision-making style [27]. Emotionally intelligent people may prompt others to act rationally like they themselves usually do so. The other possibility is that emotionally intelligent people attempt to regulate others' emotions based on their own goals. Because EI just reflects how well individuals process emotions and emotional information, emotionally intelligent people are not necessarily attempting to achieve general prosocial outcomes. Recent studies suggested that emotionally intelligent people may regulate emotions to accomplish their own goals rather than to achieve general prosocial outcomes [7]–[9]. Although these studies did not directly assess any specific goals in interpersonal situations or behaviors for regulating others' emotions as EI correlates, emotionally intelligent people may regulate others' emotions or ways to express their emotions based on their own goals.

It remains unclear which possibility is more plausible because previous studies have not sufficiently investigated the relationship between trait EI and behavior for regulating others' emotions as discussed above. The main purpose of this study is to reveal this point for clarifying the social functions of trait EI.

### Ostracism in which individual differences in trait EI emerge

In the current study, we focused on ostracism as an interpersonal problem because individual differences in trait EI will emerge in this situation [25]. Ostracism is defined as “being ignored and excluded, and it often occurs without excessive explanation or explicit negative attention” [28]. Given our strong need to belong [29], [30], ostracism severely and negatively affects people. Previous studies have suggested that the experience of ostracism increases such negative emotions as anger and sadness and decreases such positive emotions as joy and pleasure [31], [32]. Ostracism is also a precursor to retaliation [33]. For example, in one study, the ostracized people blasted their game partners with higher levels of aversive noise than people who were included [34]. When playing a dictator game [35], the ostracized made lower offers to the players who ostracized them than to players who included them or to new players [36].

How to interact with ostracized others who attempt retaliation could be a candidate behavioral measure for assessing the regulation of emotions in others. Williams [37] indicated that future research should investigate individual reactions or behaviors when more than one person was simultaneously ostracized. In this situation, ostracized people can comfort each other, but they might also become provocative and violent more easily and quickly [37]. Since retaliation is related to such emotions as anger or aggression, individual differences in trait EI should emerge as how ostracized individuals interact with ostracized others who attempt retaliation. Our study focuses on how to interact with ostracized others who attempt retaliation as a behavior for regulating others' emotions; this is a potential correlate of trait EI.

### Present study

The present study investigates how emotionally intelligent people interact with ostracized others who attempt retaliation in an exploratory manner. It manipulated ostracism through a ball-tossing game called Cyberball [38] that the participants played with three others. During Cyberball, the participants and one other player were ostracized by the other two players in the ostracism condition; they were included in the inclusion condition. After Cyberball, they played a recommendation game, which is a variation of the ultimatum game [38] with the same three players. They decided whether to recommend that another player, who had been ostracized in the ostracism condition, should inhibit retaliation or not. This recommendation resembles a form of emotion regulation of others called “cognitive engagement,” which is a strategy that is engaged with the target's cognitions for changing emotions or emotional behavior [20]. We also assessed the trait EI of the participants and their intentions to retaliate and investigated the relationships among them.

To clarify how the intrapersonal and interpersonal domains of trait EI function in interpersonal situations, we must investigate the relationship between trait EI and its correlates on the basis of this distinction. Because interaction with others who attempt retaliation is behavior related to the emotions of others, we predict that interpersonal EI will affect how people interact with others who attempt retaliation. We reveal whether emotionally intelligent people prompt others to act rationally or regulate other's emotions based on their own goals.

## Method

### Ethical Statement

This study was approved by the ethics committee of the Graduate School of Education, Kyoto University. All participants provided informed, written consent to participate in the experiment at the beginning of the study.

### Participants

Eighty-one university or graduate students participated in the experiment. One participant realized that the other players were controlled by a computer program and thus was excluded from the analysis. This resulted in a final sample of 80 participants. They were randomly assigned to either an ostracism condition (26 males, 14 females, mean age  = 20.5, *SD* = 2.17) or an inclusion condition (26 males, 14 females, mean age = 20.3, *SD* = 2.98), while at the same time ensuring that the conditions were matched for gender ratio.

### Procedure

Four participants who did not know each other were introduced to an experimental room and were seated in front of a computer. In some groups, only three participants gathered together. In these cases, a research assistant pretended to be a participant and participated in the experiment. They could not see each other after the experiment started due to partitions separating them.

After completing a written and verbal informed consent process, participants answered the trait EI scale. Next, participants were told that this study examined the effects of mental visualization and that they would be playing an Internet ball-tossing game on the computer. They were asked to visualize the situation, themselves, and the other players. They were also instructed to put on earmuffs during the game so that they could concentrate on playing the game. The actual purpose was to prevent the participants from hearing the sounds caused by the other participants pressing keys. After the explanation of Cyberball, participants were given the following instructions. “The experimenter has sent an additional instruction to some participants. If a message appears in the lower part of the computer screen, you are one of the participants who has received the additional instruction and should read the massage silently. This additional instruction is not compulsory. You can freely decide to obey this massage or not.” In actuality, no participants received any additional message. We set this procedure to prevent participants from suspecting why ostracism happened later. Participants started playing Cyberball in accordance with the experimenter's instruction. They engaged in the experimental session following the practice session. After finishing the experimental session, they answered the questions about manipulation check and emotions felt during Cyberball.

After answering the questions, participants were told the rules of the recommendation game. They were also told that the names and place of each player were the same as in the ball-tossing game. After the explanation, the participants were asked whether there is no question about the rule and started playing the recommendation game in accordance with the experimenter's instruction. After finishing the recommendation game, they answered the questions assessing their intentions to retaliate during the recommendation game.

When the experiment was completed, participants were thoroughly debriefed. They were told the exact purpose of the study. Importantly, they were told that other players were not participants in the same room but controlled by the computer and the ostracism/inclusion episode they experienced was bogus and randomly assigned.

### Materials

#### Cyberball

We used an online ball-tossing game called Cyberball [38] to manipulate ostracism. We used Cyberball played by four people. Participants, who did not know each other, were told that they were playing the game with three other players in the same room who were also participating in this experiment. In reality, the computer controlled the three agents involved in the game. Participants were informed that they would be represented by an animated hand at the bottom of the screen whereas the other three players would be represented by animated figures located above and to the left, right and opposite side of the participant's animated hand. When the ball was tossed to the participants, they were instructed to press “1” to throw the ball to the left player, “2” to the opposite player, and “3” to the right player by using a key board. In a practice session, participants were thrown the ball roughly one fourth of the time by the other “players” in both the ostracism and the inclusion conditions. In an experimental session, participants assigned to the ostracism condition experienced that they and the opposite player received the ball twice at the beginning of the game, and for the remaining time, never received the ball again. Participants assigned to the inclusion condition received the ball one fourth of the time from the other “players” like in the practice session. The game ended after 10 throws in the practice session and 40 throws in the experimental session. It lasted approximately five minutes.

#### Recommendation game

We developed a recommendation game based on the ultimatum game [39] to assess how people interact with others who attempt retaliation. This game was also played by four players like Cyberball. In reality, all other players were controlled by the computer. One round of the game consisted of the following procedures. First, the computer randomly assigned one player as a proposer, another player as a responder, and the other two players as recommenders. Next, the proposer decided how to divide 1000 points with the responder by choosing a multiple of 100 as the amount which the proposer would continue to hold. After that, the responder tentatively decided whether to accept or reject this offer. If the responder accepted the offer, the points were distributed in accordance with the offer. If the responder rejected the offer, neither the proposer nor responder received anything. On the basis of the responder's tentative decision, the recommenders recommended whether the responder should accept or reject the offer. Given these recommendations, the responder finally decided whether to accept or reject the offer. In accordance with this final choice, the points were distributed.

Participants took part in 12 rounds: three as the proposer, three as the responder, and six as the recommender. They were told that all decision had to be made within 15 seconds. Each player's decision was only known to players who made decisions after that in a round. Specifically, the proposer could not know any decision made by the responder or recommenders, the responder could know all decisions, and the recommender could know the proposer's decision and responder's first decision. Participants were told that the responder's second decision was not known to other players until the end of the game. They were informed that the points they received during the game were exchanged for an extra reward up to 500 yen (about US$5) in accordance with an absolute amount of the points. In actuality, all participants received 500 yen as an extra reward regardless of their decisions in addition to 500 yen as a basic reward.

We set the following situation to assess how people interacted with others who attempt retaliation. First, participants were assigned to the recommender, and the proposer (the left or right player, who was the ostracizer in the ostracism condition) offered the responder (the opposite player, who was the victim in the ostracism condition) 400 points out of 1000. This offer could be regarded as fair [40]–[42]. Next, the responder chose “reject” to this offer. This behavior could be conceived as retaliation by preventing the proposer from obtaining additional points [42]–[44]. The next participant's decision as the recommender was the target for assessment as correlates of participant's trait EI. If the participant recommended that the responder should accept the offer, this behavior would make the responder realize that accepting the offer was more rational because the offer was quite fair and the responder could increase his/her points. We used whether participants recommended that the responder should accept the offer in this situation as the behavioral measure of regulating emotions in others who attempt retaliation.

To make participants believe they played with real participants, they also completed not only rounds of the above situation but also following situations (e.g., participants were assigned to be the proposer or the responder). 1: When both participants and the opposite player were assigned to the recommender, the proposer (the left or right player) offered the responder (the right or left player, respectively) 400 points out of 1000. The responder selected “accept” for this offer. 2: When the participants and the opposite player were assigned as the recommender and proposer, respectively, the opposite player offered the responder (the left or right player) 800 points out of 1000. The responder selected “reject” for this offer. 3: When the participants and the opposite player were assigned to the responder and recommender, respectively, the proposer (the left or right player) offered the participants 400 points out of 1000. Following the participants' first decision, the opposite player recommended “reject” and the other recommender (the right or left player, respectively) recommended “accept” to the participants. 4: When the participants and the opposite player were assigned to the responder and proposer, respectively, the opposite player offered the participants 400 points out of 1000. Following the participants' first decision, the left player recommended “accept” and the right player recommended “reject” to the participants. 5: When the participants were assigned to the proposer, they first decided the offer. After that, they did not know what choice the other players had selected. How other people behaved was determined considering that the opposite player attempted to cause the left and right players to lose and the left and right players mostly behaved fairly. We also analyzed the participant behaviors in these other rounds as dependent variables, though they were not main aim of this study. We found no significant relationship between trait EI and these behavioral measures.

The order of completing these rounds was randomly assigned to each participant. The game ended after participants completed all 12 rounds. It lasted approximately 15 minutes.

#### Trait EI

We used a partially revised version of the Wong and Law Emotional Intelligence Scale (WLEIS) [45], [46] used in Nozaki [47] to assess the participants' trait EI. Nozaki [47] partially revised WLEIS because the WLEIS had been criticized for having items in “use of emotion,” one of the subscales of the WELIS, including contents that were not related to emotions, and for having no items referring to the management of others' emotions [48]. This revised scale consisted of four subscales: self-emotion appraisal (e.g., I have a good understanding of my own emotions), regulation of emotions in the self (e.g., I have good control of my own emotions), other-emotion appraisal (e.g., I have a good understanding of the emotions of people around me), and regulation of emotions in others (e.g., I am good at alleviating someone's anxiety, when they feel it). Each subscale contained four items and a 6-point Likert-type scale (1 =  totally disagree to 6 =  totally agree). This scale yields two area scores each combining two subscales scores: (i) Intrapersonal EI as the combined score of self-emotion appraisal and regulation of emotions in the self; and (ii) Interpersonal EI as the combined score of other-emotion appraisal and regulation of emotions in others. Cronbach's alpha coefficients were .81 for intrapersonal EI and .82 for interpersonal EI.

#### Manipulation check

To check whether the ostracism manipulation was successful, we asked participants to answer the following questions: “What percentage of the throws was thrown to you?” [32], “To what extent were you included by the participants during the game?” [32], and “To what extent was the opposite player included by the participants during the game?” The last two questions were rated from 1 (not at all) to 7 (very much).

#### Emotions felt during Cyberball

To measure participants' appraisal of emotions felt during Cyberball by each player, they were asked to rate their agreement with the following 12 statements: “You (the left / right / opposite player) felt anger during the game,” “You (the left / right / opposite player) felt sadness during the game,” and “You (the left / right / opposite player) felt enjoyment during the game.” All items were rated from 1 (not at all) to 7 (very much).

#### Intention to retaliate

To measure participants' intentions to retaliate during the recommendation game, they rated their agreement with the following three statements: “I tried to retaliate against those who had ostracized someone,” “I decided the choices taking account of ostracism,” “I treated both those who ostracized someone and did not equally (reversed)” (α = .76). All items were rated from 1 (not at all) to 7 (very much).

## Results

### Manipulation check

To check adequacy of manipulation, we conducted a *t* test on each rating score for the questions of the manipulation check. The results revealed that participants in the ostracism condition felt significantly less included (*M* = 2.15, *SD* = 1.03) than those in the inclusion condition (*M* = 4.33, *SD* = 1.77), *t*(78) = −6.71, *p*<.001, *d* = 1.51. Participants in the ostracism condition also reported that they received the ball tosses significantly fewer times (*M* = 10.85%, *SD* = 6.46) than those in the inclusion condition (*M* = 23.35%, *SD* = 7.56), *t*(78) = −7.95, *p*<.001, *d* = 1.78. Moreover, participants in the ostracism condition reported that the opposite player was significantly less included (*M* = 2.00, *SD* = 1.01) than those in the inclusion condition (*M* = 5.22, *SD* = 1.29), *t*(78) = 12.43, *p*<.001, *d* = 2.78. These results suggested that participants correctly perceived whether they and the opposite player were ostracized or included during Cyberball.

### Emotions felt during Cyberball

We examined the differences in the emotions felt during Cyberball between conditions to investigate whether ostracized people felt more negative and fewer positive emotions when two people were simultaneously ostracized, as when one person was ostracized (for all means and standard deviations, see Table 1). Each 2 (condition: ostracism, inclusion) ×4 (player: participants, opposite, left, right) ANOVA on anger, sadness, and enjoyment ratings revealed significant interactions, *F*(3, 234) = 7.05, *p*<.001, *η_p_* = .08, *F*(3, 234) = 8.29, *p*<.001, *η_p_* = .10, and *F*(3, 234) = 21.60, *p*<.001, *η_p_* = .22, respectively. These interactions were analyzed by conducting a *t* test on the differences between conditions within each player (see Table 1). Participants in the ostracism condition rated their own and the opposite players' anger and sadness significantly higher than those in the inclusion condition. Participants in the ostracism condition rated their own and the opposite players' enjoyment significantly lower than those in the inclusion condition. Ostracized participants rated the left players' enjoyment significantly higher than the included participants did. We found no significant differences in participant ratings of the right players' enjoyment.

**Table 1:** Means and standard deviations of emotions felt during Cyberball.

	Ostracism		Inclusion				
	(*N* = 40)		(*N* = 40)				
Variable	*M*	*SD*	*M*	*SD*	*t*(78)	*p*	*d*
Anger							
Participant	3.65	1.75	2.58	1.80	2.71	.008	0.60
Opposite player	4.48	1.62	2.56	1.45	5.54	<.001	1.24
Left player	2.48	1.40	2.30	1.29	0.58	.56	0.13
Right player	2.80	1.74	2.73	1.80	0.19	.85	0.04
Sadness							
Participant	4.25	1.88	3.10	1.88	2.74	.008	0.61
Opposite player	4.95	1.34	2.88	1.60	6.28	<.001	1.40
Left player	2.27	1.32	2.12	1.24	0.52	.60	0.19
Right player	2.35	1.25	1.92	1.23	1.53	.13	0.35
Enjoyment							
Participant	2.65	1.42	4.00	1.45	−4.20	<.001	0.94
Opposite player	2.10	1.30	4.17	1.55	−6.49	<.001	1.45
Left player	4.92	1.65	4.28	1.20	2.01	.047	0.44
Right player	4.95	1.50	4.80	1.26	0.48	.63	0.11

### Effects of trait EI on the rating of emotions felt during Cyberball

We used a hierarchical multiple regression analysis to examine the effects of trait EI on the rating of emotions felt during Cyberball. We centered all continuous predictors to reduce potential problems of multicollinearity among variables [49]. We separately examined each player's emotions (three types of emotions, four types of players; 12 variables altogether) as dependent variables in each analysis. In the first step, predictor variables were gender (female = 1, male = −1), intrapersonal EI, interpersonal EI, and condition (ostracism  = 1, inclusion  = −1). In the second step, we added Intrapersonal EI× Condition interaction, and Interpersonal EI× Condition interaction. Results revealed that the Intrapersonal EI× Condition interaction was significant on the rating of the opposite players' sadness (*B* = −0.08, *p* = .006). A simple slope analysis revealed that intrapersonal EI was positively associated with rating of the opposite players' sadness in the inclusion condition (*B* = 0.10, *p* = .019). However, they were not significantly associated in the ostracism condition (*B* = −0.06, *p* = .12). No other interaction terms or main effects of each subscale of trait EI were significant. These results suggested that trait EI was not related to the rating of emotions caused by ostracism.

### Effects of trait EI on the interaction with others who attempt retaliation

We used whether participants recommended “accept” to the opposite player who had chosen to reject the fair offer by the left or right player as the behavioral measure of interaction with others who attempt retaliation. This behavior did not significantly differ between conditions (ostracism condition, 56.25%; inclusion condition, 53.75%; Mann–Whitney *U* test, *z* = 0.32, *p* = 0.75).

We used generalized liner mixed modeling (GLMM) with the logit link function to examine the effects of trait EI on the interaction with others who attempt retaliation. The GLMM was carried out with the function “lmer” of the statistics package R 2.15.1 [50] using the package “lme4” [51]. The dependent variable was dummy code of participants' recommendation (accept  = 1, reject  = 0). The coefficients were estimated by using restricted maximum likelihood and participants were treated as a random effect. All continuous predictors were centered.

First, we examined the possibility that emotionally intelligent people may prompt others to act rationally. Gender (female  = 1, male  = −1), intrapersonal EI, interpersonal EI, condition (ostracism  = 1, inclusion  = −1), Intrapersonal EI× Condition interaction, and Interpersonal EI× Condition interaction were treated as fixed effects fitting the data. Results revealed that no independent variables were significant (*ps*>.24). They did not support the idea that emotionally intelligent people prompted others to act rationally.

**Figure 1:**
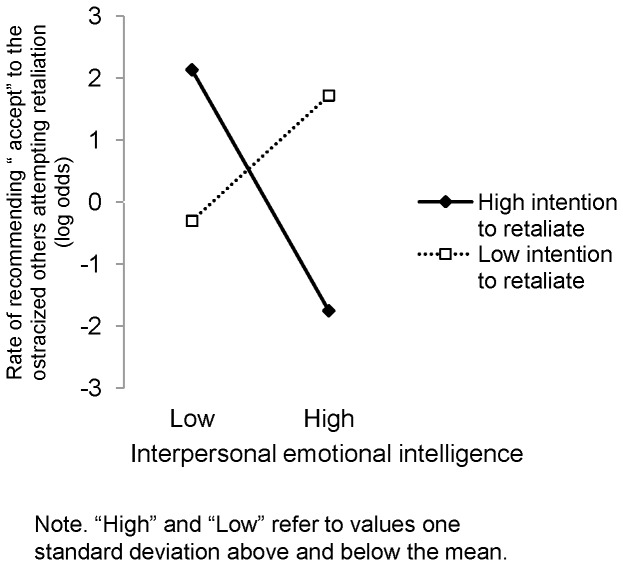
Interactive effect of interpersonal emotional intelligence and intention to retaliate. The graph illustrates the result of simple slopes for the association between interpersonal emotional intelligence and participants' recommendation to the ostracized person depending on intention to retaliate.

Second, we examined the possibility that emotionally intelligent people attempt to regulate others' emotions in accordance with their own goal. Gender (female  = 1, male  = −1), intrapersonal EI, interpersonal EI, intention to retaliate, Intrapersonal EI× Intention to retaliate interaction, and Interpersonal EI× Intention to retaliate interaction were treated as fixed effects fitting the data consisting of participants in the ostracism condition. Results revealed that the Interpersonal EI× Intention to retaliate interaction was significant (*B* = −0.06, *p* = .002). No other independent variables were significant (*ps*>.17). Following the approach recommended by Jaccard [52], we decomposed the significant interaction (Figure 1). At one standard deviation below the mean in intention to retaliate, interpersonal EI was positively related to the rate of recommending “accept” to the ostracized other player (*B* = 0.16, *p* = .04). In contrast, at one standard deviation above the mean in intention to retaliate, this association was negative and significant (*B* = −0.31, *p* = .007). These results supported the idea that emotionally intelligent people attempt to regulate others' emotions in accordance with their own goals rather than prompt others to act rationally.

## Discussion

This study, which revealed how trait EI is related to interaction with ostracized others who attempt retaliation, tested two possibilities: people with high interpersonal EI prompted others to act rationally or they attempted to regulate others' emotions based on their own goals. Our results supported the latter. People with higher interpersonal EI were more likely to recommend that the ostracized other should accept fair offers from the ostracizers when they had a weaker intention to retaliate. However, they were more likely to recommend that the ostracized other should reject fair offers when they had a stronger intention to retaliate.

### How emotionally intelligent people regulate others' emotions

The results of this study are consistent with the recent suggestion that emotionally intelligent people can regulate emotions to accomplish their own goals rather than to achieve general prosocial outcomes [7]–[9]. However, previous studies did not directly assess the goals or behaviors for regulating others' emotions in a given interpersonal situation. Therefore, this is the first laboratory study that empirically reveals that people with high interpersonal EI influence others' emotions based on their own goals.

Our study offers valuable contributions for the refinement of the trait EI concept in the respect of its social function. Trait EI has generally been associated with prosociality [3]. However, since EI just reflects how well individuals process emotions and emotional information, trait EI is not necessarily associated with prosociality. Our results suggest that researchers should consider it a personality trait that is unrelated to prosociality, contrary to its general view. Trait EI itself is neither positive nor negative, but it can facilitate interpersonal behaviors for achieving goals.

Neither intrapersonal nor interpersonal EI was significantly related to intention to retaliate of ostracized participants (*r* = .10, *p* = .54; *r* = .21, *p* = .20, respectively). In the current study, participants were not acquaintances and no interpersonal relationship continued after the recommendation game. Thus, one could regard both cooperating with ostracizers to maximize their reward by inhibiting retaliation and retaliating against ostracizers to punish them as valid behavior. For this reason, we think there were no significant relationships between trait EI and intention to retaliate. If interpersonal relationships continue after the recommendation game, trait EI may be related to the intention to retaliate depending on the social context. Ford and Tamir [53] revealed that people with high trait EI preferred to feel angry in confrontational situations, whereas they preferred to feel happy in collaborative situations. Given the results of this study, emotionally intelligent people may have stronger intentions to retaliate if a following task needs a confrontational relationship with offenders, while they have weaker intention to retaliate if a following task needs a collaborative relationship with offenders. Future research should examine when and how trait EI affects goal-setting processes by manipulating social contexts (e.g., confrontational or collaborative).

### Ostracism and trait EI

Given that EI is the subset of social intelligence [54], it is important to reveal how emotionally intelligent people behave in interpersonal situations. However, no previous study has investigated how emotionally intelligent people behave for regulating others' emotions. When people confront interpersonal problems, they have to appraise and regulate others' emotions as well as their own emotions to achieve a constructive outcome. Therefore, how people behave in an ostracism situation will reflect the individual differences in trait EI. Because previous studies on ostracism often employed laboratory studies and used behavioral measures [31], [36], [38], we believe that applying this paradigm to EI studies is good way to solve the above limitation. These kinds of studies could deepen our understanding of the social functions of trait EI.

In line with the previous study using other data [25], trait EI was not significantly related to the rating of each player's emotion during Cyberball in the ostracism condition. Emotions have a function of facilitating useful reactions in daily life. For example, anger defends people from threats around them [55] and sadness alerts people that something bad is happening to them [56]. Because negative emotions functioned adaptively right after experiencing ostracism, participants did not have to regulate negative emotions in this situation even though they had high trait EI. Furthermore, we set up the situation of ostracism by employing the Cyberball paradigm as an extreme situation in which the left and right players continued to throw the ball to each other. In this extreme situation, participants easily inferred that the other ostracized person felt more negative emotions and less positive emotions, even if participants' trait EI was not high.

We did not assess emotions at baseline in this study. Moreover, participants retrospectively reported emotions felt during Cyberball. Thus, future research should assess emotions at baseline and throughout experiencing ostracism to more rigorously examine the relationship between trait EI and rating of each player's emotion during ostracism.

### Limitations and future directions

Several limitations may prove beneficial in generating future research ideas. First, the situation in which two people were ostracized simultaneously might be regarded as a conflict between pairs of people rather than ostracism. However, corresponding to previous studies in which only one person was ostracized [31], [32], participants in the ostracism condition rated that they and the opposite player who experienced ostracism felt more anger, more sadness, and less enjoyment during Cyberball than in the inclusion condition. Moreover, the definition of ostracism does not contain isolation as a prerequisite [28]. Given that the Cyberball paradigm itself was a task for manipulating ostracism, the situation we set up could be regarded as ostracism rather than a conflict between pairs of people. To examine this point further, future research should use measures specific to ostracism such as lowering a feeling of belonging.

Second, there was no significant difference between conditions in the rate of recommending “accept” to the opposite player who had chose to reject the fair offer by the left or right player. In this situation, either recommending ostracized people to accept or to reject the offer was a valid action, because the former could maximize the ostracized others' additional reward by regulating others' anger while the latter could respect the will of the ostracized others. There was no reason to predict that people were more or less likely to recommend ostracized people to accept the offer than the included people. For this reason, we think that the rates of the “accept” recommendation did not differ between conditions. If the types of regulation of others' emotion were different from attempts to regulate retaliation such as consoling someone in sorrow or making someone laugh, people may more likely behave in such a way to ostracized others than included others. Future research could examine this possibility.

Third, it remains unclear whether trait EI still predict how to interact with ostracized others who attempt retaliation over and above established constructs such as Big-Five, because we did not assess other self-concept measures. However, given that many previous studies has demonstrated trait EI predicts a number of emotional reactions and achievements after statistically controlling for other established constructs [12]–[14], there is a high possibility that the relationship which this study revealed will still exist over and above other constructs. To clarify this point, future research should assess other established constructs (e.g., Big-Five) and examine whether trait EI still predict behavior for regulating others' emotions after statistically controlling these constructs.

Forth, various other EI measures were used in previous studies such as Trait Emotional Intelligence Questionnaire (TEIQue) [2]. Future research should investigate whether the results of this study could be replicated by using other EI measures.

### Conclusion

This is the first laboratory study to empirically reveal that those who have high interpersonal EI attempt to regulate others' emotions based on their own goals. People with higher interpersonal EI were more likely to recommend that the ostracized other should accept a fair offer from the ostracizers when the emotionally intelligent people had a lower intention to retaliate. However, they were more likely to recommend that the ostracized other should reject the offer when the emotionally intelligent people had a higher intention to retaliate. These results suggest that researchers should consider trait EI a personality trait that is not necessarily associated with prosociality, contrary to the general view. Trait EI facilitates interpersonal behaviors for achieving goals. This study presents an important step toward deepening our understanding of trait EI's social functions.
